# Efficacy and safety of rivaroxaban versus low-molecular-weight heparin for the prevention of symptomatic in-hospital venous thrombosis following primary total hip and knee arthroplasty: a large-sample, multicenter, retrospective study

**DOI:** 10.1186/s12891-026-09866-y

**Published:** 2026-06-05

**Authors:** Yuanping Liao, Jie Deng, Xiaotao Shi, Xiao Wang, Honglue Tan, Guorui Cao

**Affiliations:** 1 Luoyang Orthopedic Hospital of Henan Province, Luoyang, China, People’s Republic; 2 Guangzhou University of Chinese Medicine Dongguan Hospital, Dongguan, GuangDong Province People’s Republic of China

**Keywords:** Rivaroxaban, Low molecular weight heparin, Total hip arthroplasty, Total knee arthroplasty, Symptomatic thrombosis

## Abstract

**Purpose:**

To evaluate the efficacy and safety of rivaroxaban and low molecular weight heparin (LMWH) for the prevention of postoperative venous thrombosis (VTE) following primary unilateral total hip arthroplasty (THA) and total knee arthroplasty (TKA).

**Methods:**

A total of 11,686 patients who received rivaroxaban and LMWH (6,110 cases of THA and 5,576 cases of TKA) between January 2014 and November 2017 in 26 large teaching hospitals were enrolled. The patients were divided into two groups according to the postoperative use of anticoagulant drugs, including 4,591 cases in the rivaroxaban group (2,288 cases of THA and 2,303 cases of TKA) and 7,095 cases in the LMWH group (3,822 cases of THA and 3,273 cases of TKA). The incidence of deep vein thrombosis (DVT) in the lower extremities, and pulmonary embolism (PE), were analyzed as indicators of efficacy. The incidences of postoperative bleeding events, total blood loss (TBL), and transfusion rate, were considered as safety indicators.

**Results:**

The overall incidence of DVT was 0.40% (47/11,686) in patients undergoing primary THA/TKA. The incidence of DVT in the rivaroxaban group was 0.74% (34/4591); this was significantly higher than that in the LMWH group (0.18%; 13/7095) (OR 4.07, 95% CI 2.14–7.71;*p* < 0.001). In the THA subgroup, the incidence of DVT in the rivaroxaban group was 0.52% (12/2288 cases); this was significantly higher than that in the LMWH group 0.10% (0.10%; 4/3822) (*p* = 0.002). In the TKA subgroup, the incidence of DVT was 0.96% (22/2303 cases) in the rivaroxaban group and 0.27% (9/3273 cases) in the LMWH group (*p* < 0.001). Only one patient treated with LMWH after TKA was diagnosed with symptomatic PE in this study. There was no significance difference in terms of safety between rivaroxaban and LMWH (*p* > 0.05) in terms of the incidence of bleeding events and TBL. However, in the TKA subgroup, TBL was significantly higher in the rivaroxaban group than in the LMWH group (915 ± 514 ml vs. 872 ± 487 ml, *p* = 0.030). With regards to transfusion rate, patients in the rivaroxaban group had a significantly higher need for blood transfusion than those in the LMWH group (OR 3.30,95% CI 2.88–3.78;*p* < 0.001). Subgroup analysis revealed that the transfusion rates in the THA and TKA sub-groups were both higher in the rivaroxaban group (14.8%, 14.2%) than those in the LMWH group (6.5%, 3.0%) (*p* < 0.001).

**Conclusions:**

For patients following primary unilateral THA/TKA, LMWH is superior to rivaroxaban in terms of the prevention of symptomatic in-hospital DVT‚ and the control of TBL and transfusion rates. However, the efficacy of these drugs for controlling bleeding events was similar when compared between the two groups.

## Introduction

 Total hip arthroplasty (THA) and total knee arthroplasty (TKA) are important treatments for end-stage hip and knee disease and can provide significant pain relief and improve a patient’s quality-of-life [1]. However, postoperative venous thromboembolism (VTE) is a common complication that can arise from the procedures, including lower extremity deep vein thrombosis (DVT) and pulmonary embolism (PE). The occurrence of VTE can delay early recovery, is associated with an increased risk of thrombus dislodgement and increased mortality, and also occupies healthcare resources for a longer period of time, thus leading to a huge economic burden for the health system [2, 3]. This is particularly the case for patients with symptomatic thrombosis due to the patients complexity of this condition. In 2012, the American College of Chest Physicians published related guidelines that clearly stated that drugs, including low molecular weight heparin (LMWH), rivaroxaban, aspirin, and fondaparinux, are effective for VTE prophylaxis following THA/TKA (grade 1B) [4]. Another study reported that these medications resulted in sufficient prophylaxis, a significantly reduced risk of VTE, with a symptomatic VTE rate of only 4.3% [5]. However, there is a clear lack of a uniform consensus relating to optimal pharmacological prophylaxis, as various drugs remain controversial in terms of their efficacy and safety.

Balancing the relationship between efficacy and safety when selecting anticoagulant drugs remains a significant challenge for clinicians. Currently, thromboprophylaxis regimens for THA and TKA are generally determined according to the experience of the treating clinician. The most commonly used drugs are LMWH and rivaroxaban. Over recent decades, LMWH has been widely used in clinical practice and can be effective in reducing the risk of postoperative thrombosis. Consequently, LMWH has become the standard anticoagulant for the clinical prevention of VTE in some hospitals [6]. However, LMWH is not easy to administer and requires subcutaneous injection, thus increasing the workload of nurses and reducing patient compliance. In addition, there is a significant lack of studies relating to the safety of LMWH during the postoperative phase. Over recent years, rivaroxaban has gained popularity in an increasing number of countries and regions, largely due to the fact that it can be managed with convenience in the clinic. Moreover, large-scale clinical studies have showed that rivaroxaban has greater benefits when compared to LMWH in terms of preventing the incidence of VTE and the safety of controlling postoperative bleeding following THA/TKA [7–10]. However, some studies also reported that rivaroxaban did not provide a greater therapeutic advantage than LMWH in the prevention of VTE. Furthermore, in terms of safety, rivaroxaban is known to increase the risk of postoperative complications, including bleeding [11, 12]. Overall, the preferable choice between these two anticoagulants remains controversial. In addition, some prospective studies reported that the manner in which LMWH is administered can affect patient compliance [11] and may induce clinical distress, thus creating uncertainty when selecting between the two thromboprophylactic drugs. Due to the lack of large, multi-center studies, the selection of LMWH or rivaroxaban for postoperative thromboprophylaxis in cases of THA/TKA remains inconclusive.

At present, there is a notable lack of large data analyses comparing the efficacy and safety of these two anticoagulants following primary THA/TKA, especially in Asian regions. Previous studies mainly focused on a single center and featured a small number of samples. The present study aims to address this clear gap in the literature.

The primary objective of this study was to compare the efficacy of rivaroxaban and LMWH for postoperative symptomatic in-hospital VTE in patients following primary unilateral THA/TKA. Our secondary objective was to evaluate the safety of these two drugs, including the incidence of postoperative bleeding events, total blood loss (TBL), and transfusion rate.

## Materials and methods

### Research design and objectives

This retrospective cohort study was a sub-study conducted within a large prospective observational program sponsored by the Chinese Health Ministry, which attempted to investigate the efficacy and safety of hip and knee arthroplasty. We include patients who underwent primary THA and TKA between January 2014 and November 2017 in 26 large teaching hospitals. Ethical approval was obtained from the Biomedical Ethics Committee.

**Fig. 1:**
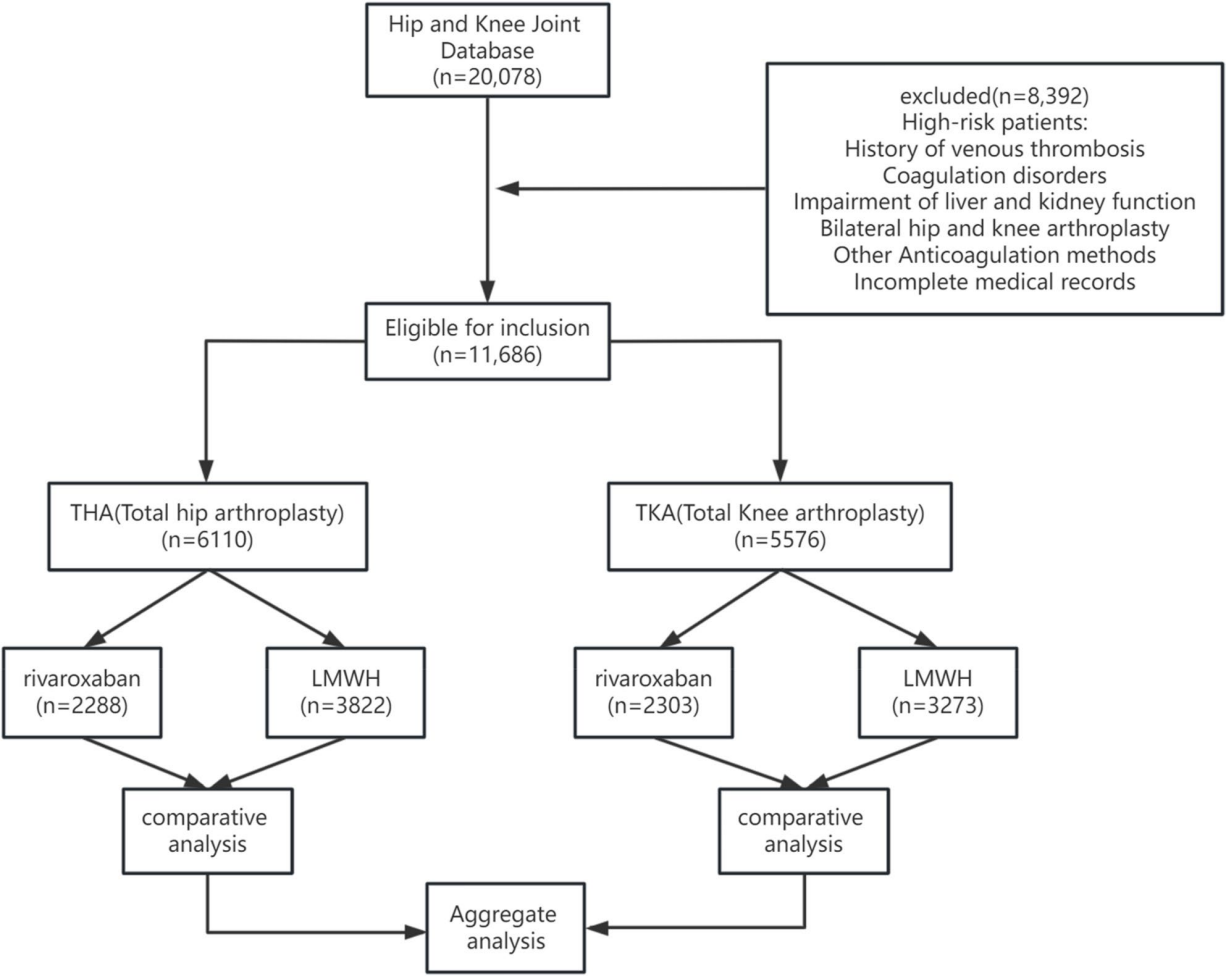
Process of sample screening and outcome. THA, total hip arthroplasty; TKA, total knee arthroplasty; LMWH, low-molecular-weight heparin

The inclusion criteria were patients ≥ 18 years-of-age who underwent primary unilateral THA/TKA and able to provide informed consent. Patients were excluded if they had predetermined high risk factors for bleeding (such as a gastrointestinal ulcer or had suffered hemorrhagic stroke), coagulation dysfunction, hepatic or renal impairment (such as levels of aspartate aminotransferase or alanine aminotransferase that were twice the upper limit of the normal range; a creatinine clearance < 30 ml/min), or if they were on anticoagulant therapy or had incomplete medical records. Patients who underwent concurrent bilateral THA/TKA or staged bilateral THA/TKA were also excluded (Fig. 1).

A total of 11,686 patients were enrolled in this study, of which 6,110 underwent THA and 5,576 underwent TKA. Patients were divided into two groups according to the anticoagulant drugs they received postoperatively. Patients in the rivaroxaban group (4591 patients) were treated with oral rivaroxaban (10 mg; Xarelto, Bayer, Germany) while patients in the LMWH group (7095 patients) were treated with subcutaneous injections of LMWH (4000U; Clexane; Sanofi-Aventis, France). The application time was 6–8 h postoperatively, repeated once a day for 14 days.All patients were suggested to received physical thromboprophylaxis measures.Physical prophylaxis included the exercises of ankle pump and knee extension when awakening from anesthesia, and the application of intermittent pneumatic compression device early postoperatively. Subgroups of the two groups were analyzed according to the type of surgery (THA/TKA). In total, 2288 patients were treated with rivaroxaban and 3822 with LMWH in the THA group. In contrast, 2303 patients were treated with rivaroxaban and 3273 with LMWH in the TKA group.

### Assessment of efficacy outcomes

The main efficacy indicators were the incidence of symptomatic in-hospital DVT and PE within 14 days. The doctors and nurses screened all enrolled patients daily for clinical signs of VTE. All patients underwent diagnostic Doppler ultrasound of both lower extremities on day 3 postoperatively. If a patient had symptoms of DVT, such as pain, swelling, and localized elevation of skin temperature in the affected extremity, then direct ultrasound or lower extremity venography were performed. Standardized compression ultrasound was used to confirm the presence of DVT. Since there is no gold standard for the diagnosis of symptomatic VTE, the diagnosis was based on clinical suspicion confirmed by venography or ultrasonography combined with impedance volume tracing [1, 13]. When PE was suspected on the basis of clinical features, such as respiration, chest pain, or tachycardia, confirmation was achieved by perfusion/ventilation imaging, pulmonary angiography, or spiral CT by a senior imaging physician.

### Assessment of safety outcomes

The main safety indicators were the incidence of postoperative bleeding events, TBL and transfusion rate. Bleeding events that occurred postoperatively were assessed based on standard definitions. Events categorized as hemorrhage from organs and requiring surgical intervention were defined as a major bleeding event, while incisional bleeding or hematomas were defined as a minor bleeding event [1]. Total blood loss(TBL) was calculated according to the Nadler and Gross formula [14]. TBL = Patient’s blood volume (PBV) × (Hctpre-Hctpost)/ Hctave. (PBV = k1 × height (m)^3^ + k2 × body weight (kg) + k3 (male k1 = 0.3669, k2 = 0.03219, k3 = 0.6041; female k1 = 0.3561, k2 = 0.03308, k3 = 0.1833. Hctpre = initial preoperative Hct level, Hctpost = postoperative Hct level on the third postoperative day, and Hctave = mean of Hctpre and Hctpost). If allogeneic transfusion was performed, then the TBL was equal to the loss calculated from the change in Hct plus the volume transfuse [14].

### Statistical analysis

All analyses were performed using SPSS version 27.0 (SPSS Inc., USA). Continuous variables are presented as mean ± standard deviation, and categorical variables as number (percentage). The normality of continuous variables was assessed using the Shapiro–Wilk test. Between-group comparisons were performed using the independent-samples t test, Wilcoxon Mann-Whitney U test, Pearson‘s chi-square test, or Fisher’s exact test, as appropriate. All reported p-values are two-sided and were not adjusted for multiple comparisons. For key outcomes, effect estimates (e.g., odds ratios, mean differences) are reported with their 95% confidence intervals (CIs). A p-value < 0.05 was considered statistically significant.

### Data handling and missing values

Analyses were conducted on the available case data. Data for the primary efficacy and safety outcomes (i.e., symptomatic in-hospital DVT, PE, bleeding events, and transfusion) were complete for all patients, with no missing values. For derived continuous variables (e.g., total blood loss), cases with missing prerequisite laboratory values (e.g., hematocrit) were excluded from the corresponding analysis (i.e., a complete-case analysis was applied). No imputation was performed for missing values.

## Results

### Study population and demographics

Of the 11,686 patients included in this study, 4,591 patients received rivaroxaban postoperatively (THA2288, TKA2303) and 7,095 patients received LMWH postoperatively (THA3822, TKA3273). The general preoperative characteristics of the two groups were assessed and there were no significant differences between the two groups in terms of baseline characteristics (Table 1).

**Table 1:** Baseline characteristics of the patients

Variables	THA			TKA		
	Rivaroxban (*n* = 2288)	LMWH (*n* = 3822)	*p* value	Rivaroxaban (*n* = 2303)	LMWH (*n* = 3273)	*p* value
Demographic characteristics						
Age, years ( *x* ± *s)*	58.0 ± 13.8	58.3 ± 13.7	0.512	66.2 ± 8.6	66.6 ± 8.7	0.055
Female gender (*n*, %)	1283(56.1%)	2052(53.7%)	0.070	1831(79.5%)	2542(77.7%)	0.100
BMI(*x* ± *s*,kg/m^2^)	23.8 ± 3.4	23.7 ± 3.5	0.401	25.8 ± 3.6	25.6 ± 3.6	0.072
Primary joint disease			0.165			0.517
OA(*n*, %)	716 (31.3%)	1302 (34.1%)		2132(92.3%)	3048(93.1%)	
ONFH(*n*, %)	721 (31.5%)	1142 (29.9%)		-	-	
DDH(*n*, %)	346 (15.1%)	592 (15.5%)		-	-	
Fracture(*n*, %)	313 (13.7%)	499 (13.1%)		-	-	
AS(*n*, %)	43 (1.9%)	52 (1.4%)		-	-	
RA(*n*, %)	61 (2.7%)	83 (2.2%)		157 (6.8%)	201 (6.1%)	
Others(*n*, %)	88 (3.8%)	152 (4.0%)		14 (0.6%)	24 (0.7%)	
Comorbidities(*n*,%)						
Hypertension	305 (13.3%)	502 (13.1%)	0.827	615(26.7%)	800 (24.4%)	0.056
Diabetes	93 (4.1%)	172 (4.5%)	0.419	202 (8.8%)	242 (7.4%)	0.061
Coronary heart disease	28 (1.2%)	54 (1.4%)	0.534	82 (3.6%)	95 (2.9%)	0.168
Surgical site(*n*,%)			0.293			0.758
Left	1117(48.8%)	1919(50.2%)		1139(49.5%)	1605(49.0%)	
Right	1171(51.2)	1903(49.8%)		1164(50.5%)	1668(51%)	

*THA* total hip arthroplasty, *TKA* total knee arthroplasty, *LMWH* low-molecular-weight heparin, *BMI* body mass index, *OA* osteoarthritis, *ONFH* osteonecrosis of femoral head, *DDH* developmental dysplasia of the hip, *AS* ankylosing spondylitis, *RA* rheumatoid arthritis

### Efficacy outcomes

**Fig. 2:**
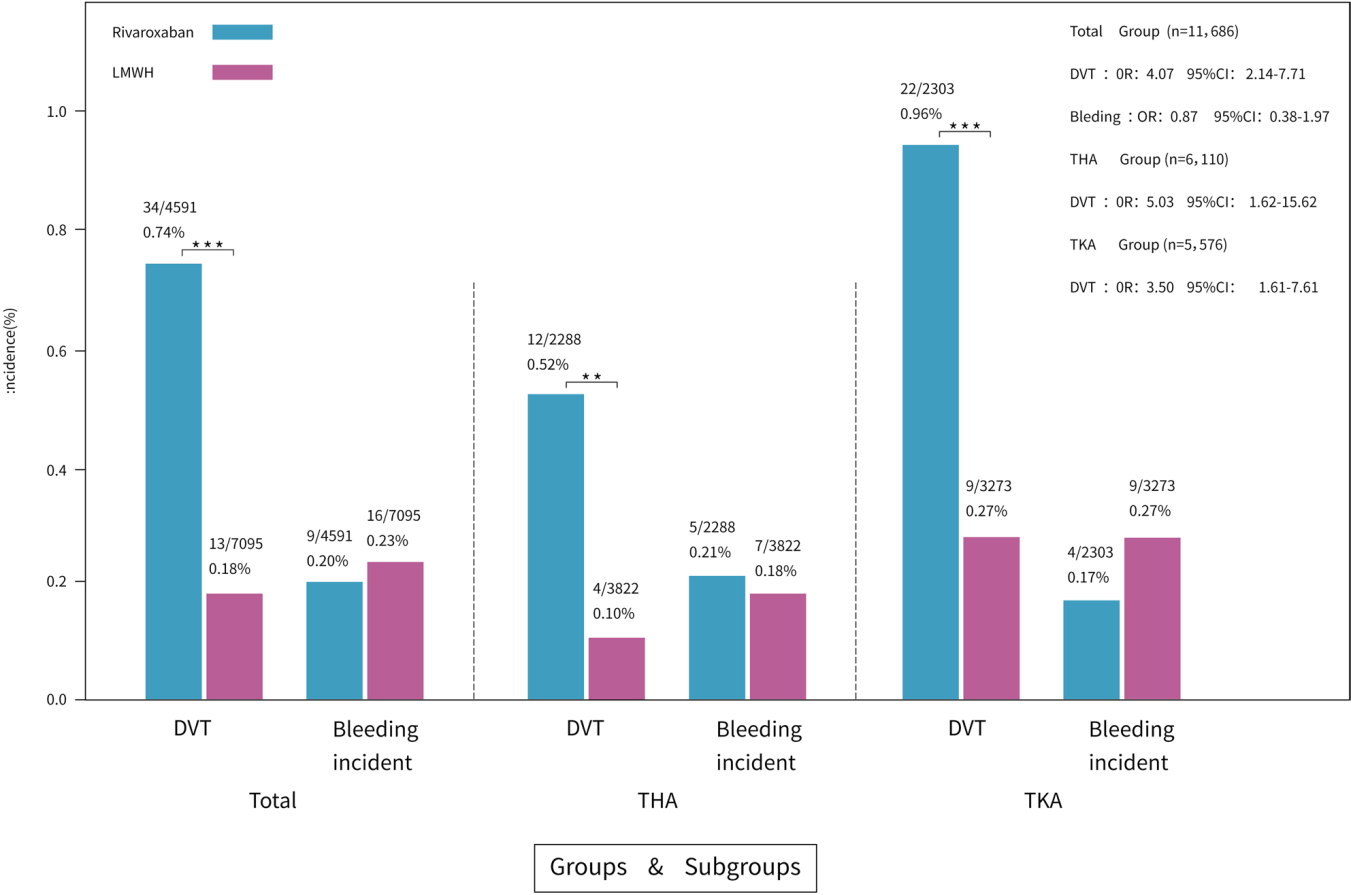
Rivaroxaban vs. LMWH: DVT & Bleeding Incident (Total/THA/TKA Subgroups). THA, total hip arthroplasty; TKA, total knee arthroplasty; LMWH, low-molecular-weight heparin. LMWH: low-molecular-weight heparin; DVT, deep vein thrombosis; THA, total hip arthroplasty; TKA, total knee arthroplasty; OR, odds ratio; CI, confidence interval; ***p<0.001, **p<0.01

DVT rates varied among patients receiving different patterns of pharmacological thromboprophylaxis (Fig. 2). The overall incidence of DVT in the lower extremities after primary THA/TKA was 0.40% (47/11686); 0.74% (34/4591) in the rivaroxaban group compared to 0.18% (13/7095) in the LMWH group (OR = 4.07, 95% CI: 2.14 to 7.71; *p* < 0.001; Table 2). THA subgroup analysis showed that the incidence of DVT was 0.52% (12/2288) in the rivaroxaban group and 0.10% (4/3822) in the LMWH group (OR = 5.03, 95% CI: 1.62 to 15.62; *p* = 0.002). TKA subgroup analysis showed that the incidence of DVT was 0.96% (22/2303) in the rivaroxaban group and 0.27% (9/3273) in the LMWH group (OR = 3.50, 95% CI: 1.61 to 7.61; *p* < 0.001)). One patient in the LMWH group developed symptomatic PE and underwent TKA (Table 3).

**Table 2:** Primary efficacy and safety outcomes

Variables	Rivaroxaban (*n* = 4591)	LMWH (*n* = 7095)	χ^2^/F值	*p* value	Effect Size (95% CI)
DVT, no. (%)	34(0.74%)	13(0.18%)	21.6	<0.001*	OR = 4.07 (2.14–7.71)
PE, no.	-	1	-	-	
Bleeding incident	9(0.20%)	16(0.23%)	0.113	0.736	OR = 0.87 (0.38–1.97)
Major bleeding event	6	7	0.257	0.612	
Minor bleeding event	3	9	0.516	0.473	
TBL(ml)	987 ± 556	971 ± 543	1.697	0.317	MD = 15.1 (-14.4–44.6)
Transfusion rate, no. (%)	666(14.5%)	347(4.9%)	325	<0.001*	OR = 3.30 (2.88–3.78)

*LMWH* low-molecular-weight heparin, *VTE* venous thrombosis, *DVT* deep venous thrombosi, *PE* pulmonary embolism *TBL* total blood loss

**Table 3:** Comparison of the efficacy of anticoagulants under THA and TKA

Variables	THA				TKA			
	Rivaroxban (*n* = 2288)	LMWH (*n* = 3822)	*p* value	Effect Size (95% CI)	Rivaroxaban (*n* = 2303)	LMWH (*n* = 3273)	*p* value	Effect Size (95% CI)
Incidence of VTE								
DVT, no.(%)	12(0.52%)	4(0.10%)	0.002*	OR = 5.03(1.62–15.62)	22 (0.96%)	9(0.27%)	<0.001*	OR = 3.50 (1.61–7.61)
PE, no.	-	-	-		-	1	-	

*THA* total hip arthroplasty, *TKA* total knee arthroplasty, *LMWH* low-molecular-weight heparin, *VTE* venous thrombosis, *DVT* deep venous thrombosis, *PE* pulmonary embolism

### Safety outcomes

The overall incidence of postoperative bleeding events in this study was 0.21% (25/11686); there was no significant difference between the two groups(OR = 0.87, 95% CI: 0.38 to 1.97; *p* = 0.736, Fig. 2)). TBL was higher in the rivaroxaban group than in the LMWH group, although this was not significant (MD = 15.1 ml, 95% CI: -14.4 to 44.6 ml; *p* = 0.317). TKA subgroup analysis showed that TBL was significantly higher in the rivaroxaban group (915 ml ± 514 ml) than in the LMWH group (872 ml ± 487 ml)(MD = 42.6 ml, 95% CI: 4.0 to 81.2 ml; *p* = 0.030); however, this was not significant in the THA group (MD = 33.7 ml, 95% CI: -10.6 to 78.0 ml; *p* = 0.145). With regards to the transfusion rate, 14.5% and 4.9% of patients in the rivaroxaban group and LMWH group required a blood transfusion, respectively (OR = 3.30, 95% CI: 2.88 to 3.78; *p* < 0.001)). Subgroup analysis showed that the transfusion rate for both THA and TKA was higher in the rivaroxaban group (14.8%, 14.2%) than in the LMWH group (6.5%, 3.0%) (OR = 2.50, 95% CI: 2.10 to 2.97; *p* < 0.001;OR = 5.32, 95% CI: 4.22 to 6.71; *p* < 0.001)). These results are summarized in Tables 2 and 4.

**Table 4:** Comparison of the safety of anticoagulants under THA and TKA

	THA				TKA			
	Rivaroxban (*n* = 2288)	LMWH (*n* = 3822)	*P* value	Effect Size (95% CI)	Rivaroxaban (*n* = 2303)	LMWH (*n* = 3273)	*P* value	Effect Size (95% CI)
TBL	1077 ± 593	1044 ± 570	0.140	MD = 33.7(-10.6-78.0)ml	915 ± 514	872 ± 487	0.030*	MD = 42.6 (4.0 -81.2)
transfusion rate	338(14.8%)	248(6.5%)	<0.001*	OR = 2.50 (2.10–2.97)	328(14.2%)	99(3.0%)	<0.001*	OR = 5.32 (4.22–6.71)
Bleeding incident	5(0.21%)	7(0.18%)	0.997		4(0.17%)	9(0.27%)	0.440	
Major bleeding	3	2	0.562		3	5	1.000	
Minor bleeding	2	5	0.925		1	4	0.608	

*THA* total hip arthroplasty, *TKA* total knee arthroplasty, *LMWH* low-molecular-weight heparin, *TBL* total blood loss, *PBV* parenchymal blood volume

### Sensitivity analysis

To quantify the robustness of our primary findings to potential unmeasured confounding, we calculated E-values. For the significantly increased risk of symptomatic in-hospital DVT associated with rivaroxaban (OR = 4.07, 95% CI: 2.14 to 7.71), the E-value was 7.3 for the point estimate, and 3.3 for the lower confidence limit. Regarding the increased risk of transfusion (OR = 3.30, 95% CI: 2.88 to 3.78), the corresponding E-values were 5.8 and 5.1, respectively. In the TKA subgroup, the markedly higher transfusion risk (OR = 5.32, 95% CI: 4.22 to 6.71) had an E-value of 9.0 for the point estimate and 7.1 for the lower CI limit. These results indicate that an unmeasured confounder would need to be strongly associated with both the choice of anticoagulant and the outcome, with risk ratios ranging from 3.3 to 9.0, to fully explain away these observed associations, suggesting that the findings are robust to potential confounding.

## Discussion

Lower extremity arthroplasty, predominantly THA and TKA, carries the risk of postoperative VTE. However, the selection of postoperative medications to prevent VTE remains a key postoperative issue. Although the American College of Chest Physicians guidelines help clinicians with formulating preventive strategies, the best options following primary THA/TKA still remains limited. In addition, due to the lack of large national studies, it has become a major challenge for orthopedic surgeons to select drugs for the prevention of VTE and avoid associated postoperative complications.

Our study was a large study involving multiple teaching hospitals across the country, and used a large sample size to directly compare the differences in efficacy and safety between rivaroxaban and LMWH for the prevention of VTE in primary THA/TKA. We concluded that the incidence of postoperative VTE following arthroplasty under standardized prophylaxis was very low (< 1%), and that LMWH was more effective than rivaroxaban in preventing symptomatic in-hospital VTE, with rivaroxaban use associated with a more than four-fold increase in the odds of DVT (OR = 4.07, 95% CI: 2.14 to 7.71).This result differed from previous studies in which several large trials reported that the incidence of VTE in 5,077 patients treated with rivaroxaban after hip and knee arthroplasty was 3.3%. This was lower than the 6.1% observed in the LMWH group of patients, thus demonstrating that the efficacy of rivaroxaban for preventing postoperative VTE in patients with THA/TKA was superior to that of LMWH across the board [7, 8]. However, the most important finding of our current study was that LMWH was superior to rivaroxaban in controlling the incidence of symptomatic in-hospital DVT. This was supported by three factors: First, in a large multicenter retrospective study, Perez et al. evaluated VTE prevention in 72,670 arthroplasty patients and found that LMWH was more advantageous in preventing the incidence of postoperative VTE when compared to rivaroxaban [15], a finding that was consistent with our present study. Furthermore, several previous studies showed that LMWH as effective as rivaroxaban in preventing VTE [16, 17].This suggests that rivaroxaban is not as absolutely superior to low molecular heparin sodium as has been described in the literature. Thus, our findings were justified, although controversy remains. Second, the context of our present study was different from that of previous studies. We only analyzed the efficacy of the two drugs in preventing symptomatic in-hospital VTE under primary unilateral arthroplasty, and excluded patients with simultaneous bilateral, revision, and asymptomatic VTE. Furthermore, we only monitored thromboprophylaxis over a 14-day period in Asian patients. The application of a long follow-up visit can be prone to the loss of data. Previous studies, which included asymptomatic VTE patients in their analysis, did not consider the incidence of patients with symptomatic VTE as the primary efficacy comparison. In addition, these studies chose to consider venous thrombosis for 30 days, or even longer, after pharmacological prophylaxis [2, 7, 8, 10, 18–20]. However, due to the different nature of the two drugs, patients with subcutaneous LMWH may have difficulty completing the entire anticoagulation regimen, this may result in a higher rate of blood clots in patients treated with LMWH and lead to a possible clinical underestimation of the efficacy of LMWH. Finally, the low incidence of VTE could have compromised the true efficacy of thromboprophylaxis. The results of a previous high-quality randomized clinical trial and observational study showed that the incidence of symptomatic DVT after THA and TKA was only 0.26% and 0.63%, respectively [13]. The incidence of symptomatic VTE in the present study was also only 0.40%, thus suggesting that VTE was uncommon. This made it difficult for the previous study to obtain sufficient statistics to identify a difference between the two drugs. In the present study, on the other hand, there were more patients using LMWH, both in the whole cohort and in the subgroup analysis, and although this was a choice of prevention taken by the physicians based on their clinical judgment, the results were more in favor of the LMWH group. However, our data did show that LMWH is more favored by clinicians in Asia. Further, high-quality studies are now needed to confirm the true efficacy of these two drugs.

Postoperative bleeding events were the main safety indictors in the present study. We found no significant difference between the two drug groups (both in terms of major and minor bleeding, OR = 0.87, 95% CI: 0.38 to 1.97). This were consistent with previous studies [18, 21]. This finding was also consistent with the results of the four RECORD trials [7–10]. There were also some inconsistencies between the reported bleeding events of some earlier studies and the present study. However, these earlier studies involved small and single-center investigations with limited clinical statistical evidence [16].

In addition to our statistics relating to the incidence of bleeding events, we also used TBL and transfusion rates to evaluate the safety of these medications. Our study showed that TBL was higher with rivaroxaban than that with LMWH, and this difference was significant in the TKA subgroup (mean difference = 42.6 ml, 95% CI: 4.0 to 81.2 ml). The rational explanation for this result is that LMWH can effectively inhibit the activation of thrombin and ensure the normal flow of blood, thus reducing postoperative blood loss without increasing the risk of bleeding [22]. A previous study reported that rivaroxaban may induce increased levels of bleeding in drug prophylaxis [23]. However, there is a clear lack of high-quality studies comparing TBL between these two drugs. Excessive postoperative blood loss is known to increase the surgical risk of arthroplasty and the incidence of complications [24]; therefore, we performed our analysis to provide reference guidelines for doctors and patients. Because rivaroxaban is associated with a higher risk of bleeding, there is also a significantly greater need for transfusion when using it (Total OR = 3.30,95% CI: 2.88 to 3.78;TKA, OR = 5.32, 95% CI: 4.22 to 6.71). A previous meta-analysis that included eight studies and a total of 13,384 patients, concluded that there was a higher transfusion requirement with rivaroxaban compared to other anticoagulants(*p* = 0.03) [18].It is also worth noting that the high rate of blood transfusion in our study was due to the lack of a consensus on standardized blood management in China at that time. We believe that there may be a greater advantage when utilizing LMWH in the postoperative management of patients with bleeding tendencies because pharmacopeial monographs provide specifications to ensure the efficacy of LMWH in relation to blood loss [25]. However, rivaroxaban lacks an effective clinical reversal agent in the event of hemorrhage.

This study also had some limitations that need to be considered.Although residual confounding from unmeasured factors cannot be ruled out in an observational study, a sensitivity analysis using E-values suggests our primary conclusions are robust. To fully explain away the increased risk of symptomatic DVT associated with rivaroxaban (OR = 4.07), an unmeasured confounder would need to be associated with both treatment allocation and the outcome by risk ratios of 7.3-fold each. This indicates that our key findings are unlikely to be negated by plausible levels of unmeasured confounding. First, because of the inherent limitations of data acquired from a large sample, multicenter, and nationwide study, there was a clear lack of clinically relevant data, including surgical procedures, implant information, and postoperative medications for rehabilitation. In addition, although standard inclusion and exclusion criteria were used, we could not adjust for all confounding factors. Second, We focused only on the incidence of in-hospital venous thromboembolism (VTE) within 14 days postoperatively, as this is the primary period of concern for VTE prevention. However, we overlooked VTE events occurring after discharge (out-of-hospital VTE), which may contribute to a higher overall incidence. Future studies will extend the follow-up period and conduct comprehensive data collection to gain a more thorough understanding of VTE incidence in both in-hospital and out-of-hospital settings. Third, we only studied the efficacy of rivaroxaban and LMWH in preventing postoperative symptomatic in-hospital VTE. Our findings may not be applicable to other thromboprophylactic agents because of the different mechanisms by which these agents prevent VTE.

Our current study determined the precise incidence of symptomatic in-hospital VTE after THA and TKA with rivaroxaban and LMWH. To our knowledge, this represents the first multicenter and large sample study in Asia to directly compare the efficacy and safety of rivaroxaban with LMWH in the prevention of symptomatic in-hospital VTE following primary unilateral arthroplasty. We utilized a more standard diagnostic approach and the fact that our patients were recruited in-hospital ensured the authenticity of the data. In the present study, the incidence of symptomatic in-hospital VTE was higher for rivaroxaban. In this regard, we cautiously recommend LMWH as the drug choice for thromboprophylaxis, although this needs to be confirmed by high-quality prospective studies in the future. The choice of prophylactic drugs still remains controversial, and there is a clear need to improve the thrombotic risk stratification system to understand how we might better decide which type of venous thromboembolic drugs are most appropriate. Recommending an individualized prophylactic regimen that balances efficacy and safety may be the way forward.

## Conclusion

In this large-scale, multicenter retrospective study of Asian patients undergoing primary THA or TKA, LMWH demonstrated a superior risk-benefit profile compared to rivaroxaban for in-hospital thromboprophylaxis. Specifically, LMWH was significantly more effective in preventing symptomatic in-hospital DVT, with rivaroxaban associated with a > 4-fold increased odds (OR = 4.07, 95% CI: 2.14 to 7.71). Regarding safety, while major bleeding rates were comparable, the use of rivaroxaban was linked to a significantly higher transfusion requirement (OR = 3.30, 95% CI: 2.88 to 3.78), a risk that was especially pronounced following TKA (OR = 5.32, 95% CI: 4.22 to 6.71). The differential impact on blood loss between THA and TKA subgroups further suggests that the type of arthroplasty is an important consideration in anticoagulant selection. These findings, supported by sensitivity analyses for unmeasured confounding, provide robust real-world evidence that challenges the assumed universal superiority of direct oral anticoagulants in this setting. For perioperative thromboprophylaxis in primary joint arthroplasty—particularly in TKA patients—LMWH appears to offer a more favorable balance of efficacy (preventing VTE) and safety (conserving blood). We therefore recommend that LMWH be considered the preferred agent in clinical protocols for this population, pending confirmation from prospective, randomized trials designed to assess patient-important outcomes like transfusion and clinically relevant blood loss.

## Data Availability

The datasets generated and/or analyzed during the current study are not publicly available due data sharing was not included in the informed consent process, but are available from the corresponding author on reasonable request.

## References

[1] Zeng Y, Si H, Wu Y, Yang J, Zhou Z, Kang P, Pei F, Shen B. The incidence of symptomatic in-hospital VTEs in Asian patients undergoing joint arthroplasty was low: a prospective, multicenter, 17,660-patient-enrolled cohort study. Knee Surg Sports Traumatol Arthrosc. 2018;27:1075–82. 10.1007/s00167-018-5253-3. 30386998 10.1007/s00167-018-5253-3

[2] Ren Y, Cao S-L, Li Z, Luo T, Feng B, Weng X-S. Comparable efficacy of 100 mg aspirin twice daily and rivaroxaban for venous thromboembolism prophylaxis following primary total hip arthroplasty: a randomized controlled trial. Chin Med J. 2021;134:164–72. 10.1097/cm9.0000000000001305. 33410616 10.1097/CM9.0000000000001305 PMC7817327

[3] Ning G-Z, Kan S-L, Chen L-X, Shangguan L, Feng S-Q, Zhou Y. Rivaroxaban for thromboprophylaxis after total hip or knee arthroplasty: a meta-analysis with trial sequential analysis of randomized controlled trials. Sci Rep. 2016;6. 10.1038/srep23726. 10.1038/srep23726 PMC4810418 27020475

[4] Kearon C, Akl EA, Comerota AJ, Prandoni P, Bounameaux H, Goldhaber SZ, Nelson ME, Wells PS, Gould MK, Dentali F, Crowther M, Kahn SR. Antithrombotic Therapy for VTE Disease. Chest. 2012;141:eS419–96. 10.1378/chest.11-2301. 10.1378/chest.11-2301 PMC3278049 22315268

[5] Xia Z-N, Zhou Q, Zhu W, Weng X-S. Low molecular weight heparin for the prevention of deep venous thrombosis after total knee arthroplasty: A systematic review and meta-analysis. Int J Surg. 2018;54:265–75. 10.1016/j.ijsu.2018.04.059. 29733996 10.1016/j.ijsu.2018.04.059

[6] Lazo-Langner A, Fleet JL, McArthur E, Garg AX. Rivaroxaban vs. low molecular weight heparin for the prevention of venous thromboembolism after hip or knee arthroplasty: a cohort study. J Thromb Haemost. 2014;12:1626–35. 10.1111/jth.12675. 25069387 10.1111/jth.12675

[7] Eriksson BI, Borris LC, Friedman RJ, Haas S, Huisman MV, Kakkar AK, Bandel TJ, Beckmann H, Muehlhofer E, Misselwitz F. W. Geerts(2008)Rivaroxaban versus Enoxaparin for Thromboprophylaxis after Hip Arthroplasty, New England Journal of Medicine358(26). 10.1056/NEJMoa0800374 18579811

[8] Turpie AGG, Lassen MR, Davidson BL, Bauer KA, Gent M, Kwong LM, Cushner FD, Lotke PA, Berkowitz SD, Bandel TJ, Benson A, Misselwitz F, Fisher WD. Rivaroxaban versus enoxaparin for thromboprophylaxis after total knee arthroplasty (RECORD4): a randomised trial. Lancet. 2009;373:1673–80. 10.1016/s0140-6736(09)60734-0. 19411100 10.1016/S0140-6736(09)60734-0

[9] Kakkar AK, Brenner B, Dahl OE, Eriksson BI, Mouret P, Muntz J, Soglian AG, Pap AF, Misselwitz F, Haas S. (2008). RECORD2 Investigators. Extended duration rivaroxaban versus short-term enoxaparin for the prevention of venous thromboembolism after total hip arthroplasty: a double-blind, randomised controlled trial. Lancet. Jul 5;372(9632):31 – 9. 10.1016/S0140-6736(08)60880-6 18582928

[10] Lassen MR, Ageno W, Borris LC, Lieberman JR, Rosencher N, Bandel TJ, Misselwitz F, Turpie AGG. Rivaroxaban versus Enoxaparin for Thromboprophylaxis after Total Knee Arthroplasty. N Engl J Med. 2008;358:2776–86. 10.1056/NEJMoa076016. 18579812 10.1056/NEJMoa076016

[11] Kim D-W, Lim S-J, Moon Y-W, Kim S-M, Park Y-S. Effect of oral factor Xa inhibitor and low-molecular-weight heparin on surgical complications following total hip arthroplasty. Thromb Haemost. 2018;115:600–7. 10.1160/th15-07-0527. 10.1160/TH15-07-0527 26790579

[12] Ricket AL, Stewart DW, Wood RC, Cornett L, Odle B, Cluck D, Freshour J, El-Bazouni H. Comparison of Postoperative Bleeding in Total Hip and Knee Arthroplasty Patients Receiving Rivaroxaban or Enoxaparin. Ann Pharmacother. 2016;50:270–5. 10.1177/1060028015626435. 26783354 10.1177/1060028015626435

[13] Januel J-M, Chen G, Ruffieux C, Quan H, Douketis JD, Crowther MA, Colin C, Ghali WA, Burnand B. f.t. Imecchi Group(2012), Symptomatic In-Hospital Deep Vein Thrombosis and Pulmonary Embolism Following Hip and Knee Arthroplasty Among Patients Receiving Recommended Prophylaxis, Jama 307(3). 10.1001/jama.2011.2029 22253396

[14] Cao G, Chen G, Huang Q, Huang Z, Alexander PG, Lin H, Xu H, Zhou Z, Pei F. The efficacy and safety of tranexamic acid for reducing blood loss following simultaneous bilateral total knee arthroplasty: a multicenter retrospective study. BMC Musculoskelet Disord. 2019;20. 10.1186/s12891-019-2692-z. 10.1186/s12891-019-2692-z PMC6626401 31299945

[15] Agaba P, Kildow BJ, Dhotar H, Seyler TM, Bolognesi M. Comparison of postoperative complications after total hip arthroplasty among patients receiving aspirin, enoxaparin, warfarin, and factor Xa inhibitors. J Orthop. 2017;14:537–43. 10.1016/j.jor.2017.08.002. 28878512 10.1016/j.jor.2017.08.002 PMC5574820

[16] Piple AS, Wang JC, Kang HP, Mills ES, Mayfield CK, Lieberman JR, Christ AB, Heckmann ND. Safety and Efficacy of Rivaroxaban in Primary Total Hip and Knee Arthroplasty. J Arthroplast. 2023;38:1613–e16201614. 10.1016/j.arth.2023.02.028. 10.1016/j.arth.2023.02.028 36805121

[17] Charters MA, Frisch NB, Wessell NM, Dobson C, Les CM, Silverton CD. Rivaroxaban Versus Enoxaparin for Venous Thromboembolism Prophylaxis after Hip and Knee Arthroplasty. J Arthroplast. 2015;30:1277–80. 10.1016/j.arth.2015.02.009. 10.1016/j.arth.2015.02.009 25724111

[18] Xu J, Chang D, Chui J, Cao J, Negus J. The efficacy and cost-effectiveness of enoxaparin versus rivaroxaban in the prevention of venous thromboembolism following total hip or knee arthroplasty: A meta-analysis. J Orthop. 2022;30:1–6. 10.1016/j.jor.2022.02.003. 35210718 10.1016/j.jor.2022.02.003 PMC8844751

[19] Hur M, Park S-K, Koo C-H, Jung ED, Kang P, Kim WH, Kim J-T, Jung C-W, Bahk J-H. Comparative efficacy and safety of anticoagulants for prevention of venous thromboembolism after hip and knee arthroplasty. Acta Orthop. 2017;88:634–41. 10.1080/17453674.2017.1361131. 28787226 10.1080/17453674.2017.1361131 PMC5694808

[20] Zou Y, Tian S, Wang Y, Sun K. Administering aspirin, rivaroxaban and low-molecular-weight heparin to prevent deep venous thrombosis after total knee arthroplasty. Blood Coagul Fibrinolysis. 2014;25(7):660–4. 24695091 10.1097/MBC.0000000000000121

[21] Long A, Zhang L, Zhang Y, Jiang B, Mao Z, Li H, Zhang S, Xie Z, Tang P. Efficacy and safety of rivaroxaban versus low-molecular-weight heparin therapy in patients with lower limb fractures. J Thromb Thrombolysis. 2014;38:299–305. 10.1007/s11239-013-1046-1. 24402194 10.1007/s11239-013-1046-1

[22] Yang T, Liu Z, Zhang B, Zhang J, Ma A, Cao D, Chen D. Comparison of the efficacy of low-molecular-weight heparin and fondaparinux sodium after total knee arthroplasty: a retrospective cohort study. BMC Musculoskelet Disord. 2023;24. 10.1186/s12891-023-06674-6. 10.1186/s12891-023-06674-6 PMC10320998 37403062

[23] Kvasnicka T, Malikova I, Zenahlikova Z, Kettnerova K, Brzezkova R, Zima T, Ulrych J, Briza J, Netuka I, Kvasnicka J. Rivaroxaban - Metabolism, Pharmacologic Properties and Drug Interactions. Curr Drug Metab. 2017;18. 10.2174/1389200218666170518165443. 10.2174/1389200218666170518165443 28524005

[24] Eichinger J, White C, Friedman R. Minimizing Blood Loss and Transfusions in Total Knee Arthroplasty. J Knee Surg. 2018;31:594–9. 10.1055/s-0038-1648223. 29727870 10.1055/s-0038-1648223

[25] Hogwood J, Mulloy B, Lever R, Gray E, Page CP, Daws L. Pharmacology of Heparin and Related Drugs: An Update. Pharmacol Rev. 2023;75:328–79. 10.1124/pharmrev.122.000684. 36792365 10.1124/pharmrev.122.000684

